# New intragastric sleeve technique reduces adipose tissue in pig experimental model: tomographic study

**DOI:** 10.1038/s41598-020-74846-8

**Published:** 2020-10-22

**Authors:** Mariana da Silva Ribeiro, Ricardo Paiva Araújo dos Scheiba Zorron, Saulo José Quina Silva, Silvia Marcela Ruiz Cadena, Marcelo Borges dos Santos Junior, Fernanda Antunes, Guilherme de Souza Vieira, Celia Raquel Quirino, André Lacerda de Abreu Oliveira

**Affiliations:** 1 Laboratory of Animal Medicine and Surgery, State University of Northern Rio de Janeiro, Campos dos Goytacazes, RJ Brazil; 2 Division of Innovative Surgery, Department of Surgery, Klinikum Bremerhaven Reinkenheide, Postbrookstraße 103, 27574 Bremerhaven, Germany; 3 Laboratory of Animal Reproduction and Genetic Improvement, State University of Northern Rio de Janeiro, Campos dos Goytacazes, RJ Brazil

**Keywords:** Obesity, Stomach

## Abstract

In order to implement a new bariatric surgery technique, we verify the efficacy of intragastric sleeve to reduce weight gain and subcutaneous adipose tissue (SAT). Animals were divided into two groups: G1 (single-port intragastric sleeve) and G2 (sham group). The stomach was surgically reduced by single-port intragastric sutures to fo a gastric sleeve. Animals were submitted to computer tomography (CT) before the surgical procedure and after 18 weeks. Images were analyzed and measurements of the thickness of SAT, depth and width of the *longissimus dorsi* muscle and the rib eye area were made. Body weight and CT measurements were analyzed using the GLM PROC. The correlation coefficients were calculated among weight, moments and measures. There was a significant difference in weight gain, in which G1 had an average of 42.803 ± 3.206 kg, lower than G2 (45.966 ± 4.767 kg). The mean values for SAT and muscle measurements differed significantly between groups, in which G1 achieved the lowest values. All variables had significant correlations and high magnitude. Intragastric *sleeve* surgery induced a significant decrease of SAT. The new intragastric *sleeve* technique is feasible, safe and effective, mainly in reducing fat deposition, making it an important alternative in bariatric surgical treatment.

## Introduction

There are several surgical techniques to treat morbid obesity. Recently, the advancement of surgical instruments has enabled the development of new surgical procedures, with emphasis on minimally invasive techniques such as laparoscopic and single-portal surgeries, which are gaining considerable interest^1–4^. The concept of single-port surgery has also found application in intragastric surgery.

The current development of minimally invasive surgery, such as intraluminal and natural orifice surgeries, is limited by issues of access, tissue manipulation and approximation^5^. The transluminal (intragastric) surgery refers to an operation performed inside the peritoneal cavity, which is accessed through a hollow viscosity (stomach)^6^. The intragastric placement of single-port devices has been described by some authors^7–9^, with emphasis on the new technique of intragastric sleeve by endoplication, which produces a significant reduction in gastric volume^10^.

The assessment and recognition of the importance of body fat distribution has led to a variety of more sophisticated methods to assess adipose tissue, such as computed tomography (CT), which is considered the reference method^11,12^. It is important to measure the adipose tissue to evaluate the results of bariatric surgery. Thus, the potential of single-port intragastric sleeve technique by endoplication in reduction of weight gain and of subcutaneous adipose tissue was evaluated in two experimental groups during 18 weeks after surgery.

## Methods

The research was approved by the Committee on Ethical Use of Animals of The State University of Northern Rio de Janeiro (protocol 765311) and were in accordance with the ethical standards of the institution. The experiment was carried out at the Animal Experimentation Unit (UEA) in 10 healthy pigs, 75 days old, weighing between 33 and 45 kg.

### Study design

Animals were divided into experimental group (G1), which was submitted to single-port intragastric sleeve surgery by endoplication (IGS-IGP); and the sham group (G2), submitted to celiotomy followed by gastrostomy. Throughout the experiment, both groups were given balanced commercial feed and water ad libitum. The postoperative survival was evaluated during 18 weeks, in which animals were weighted weekly during 18 weeks.

### Computed tomography

Animals were submitted to CT (GE LightSpeed PRO 32-slice scanner) and were placed in sternum decubitus with anterior limbs stretched above their heads to evaluate subcutaneous adipose tissue thickness (SAT), width (WLM) and depth (DLM) of the longissimus dorsis muscle, and loin eye area (LEA), measured above the last rib. This evaluation was performed in two moments: one day before surgery and on the day of death of the animals.

### Surgical technique

The pigs were submitted to 16 h of fasting. During the preoperative period, they were sedated with an intramuscular association of 2 mg kg^−1^ of acepromazine (Acepran® 1%) and 0.1 mg kg^−1^ of midazolam (Dormire®). After establishing intravenous fluid therapy, anesthesia was induced with 1 mg kg^−1^ of sodium thiopental (Thiopentax®) and 10 mg mL^−1^ propofol (Provive® 1%). The anesthesia was maintained with isoflurane (Isoforine®) and oxygen.

In both groups, the animals were placed in dorsal decubitus position and a wide trichotomy of the entire ventral abdominal region was performed followed by surgical antisepsis.

**Figure 1:**
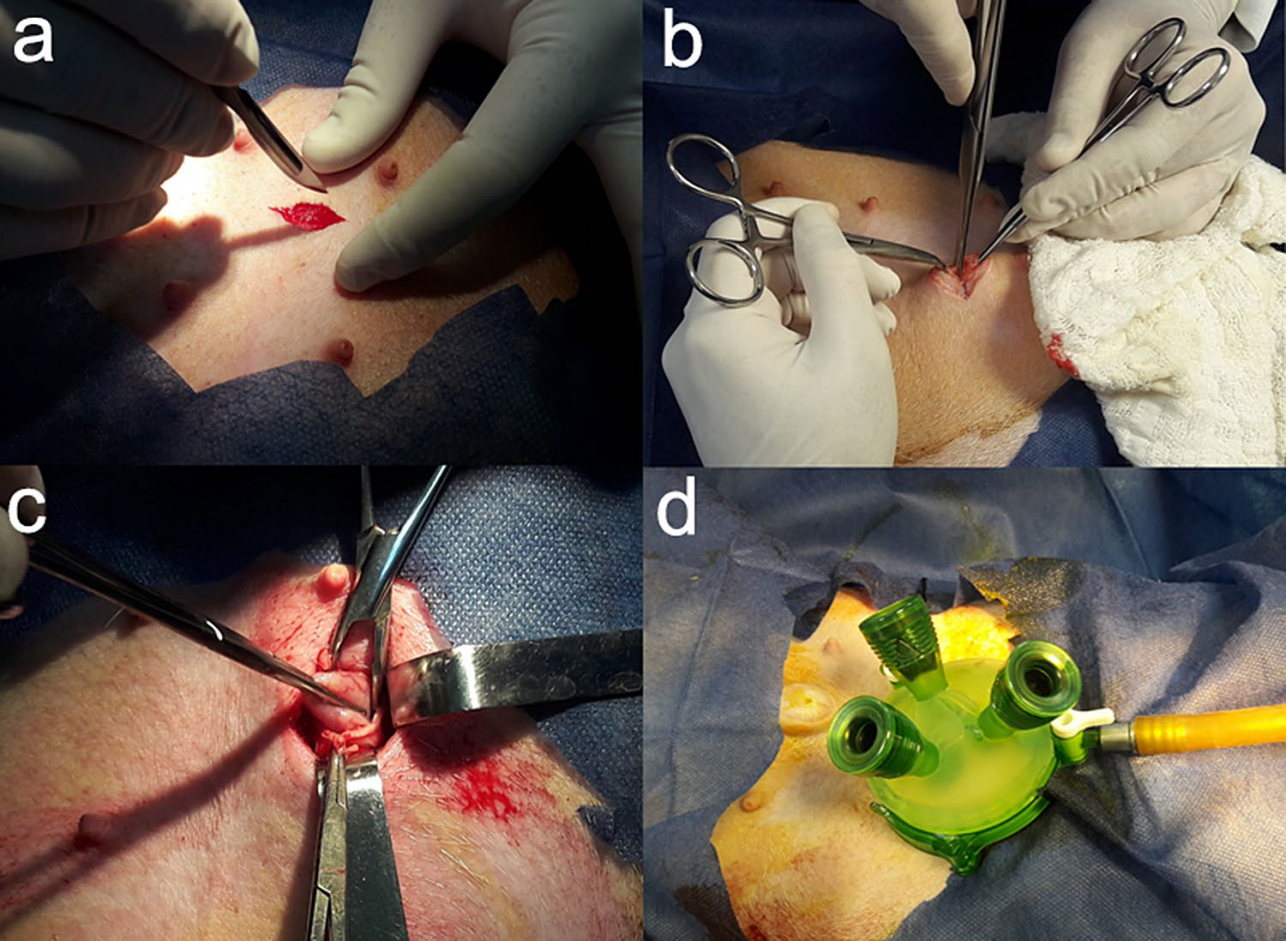
Single-port intragastric sleeve technique in pig. (**a**) Left pre-umbilical paramedian celiotomy between the cranial and caudal thoracic mammary glands. (**b**) Accessing the abdominal cavity. (**c**) Stomach seized in the body region. (**d**) a gel portal system with three working portals was introduced (GelPoint, Applied Med. RS Margarita, CA, USA).

**Figure 2:**
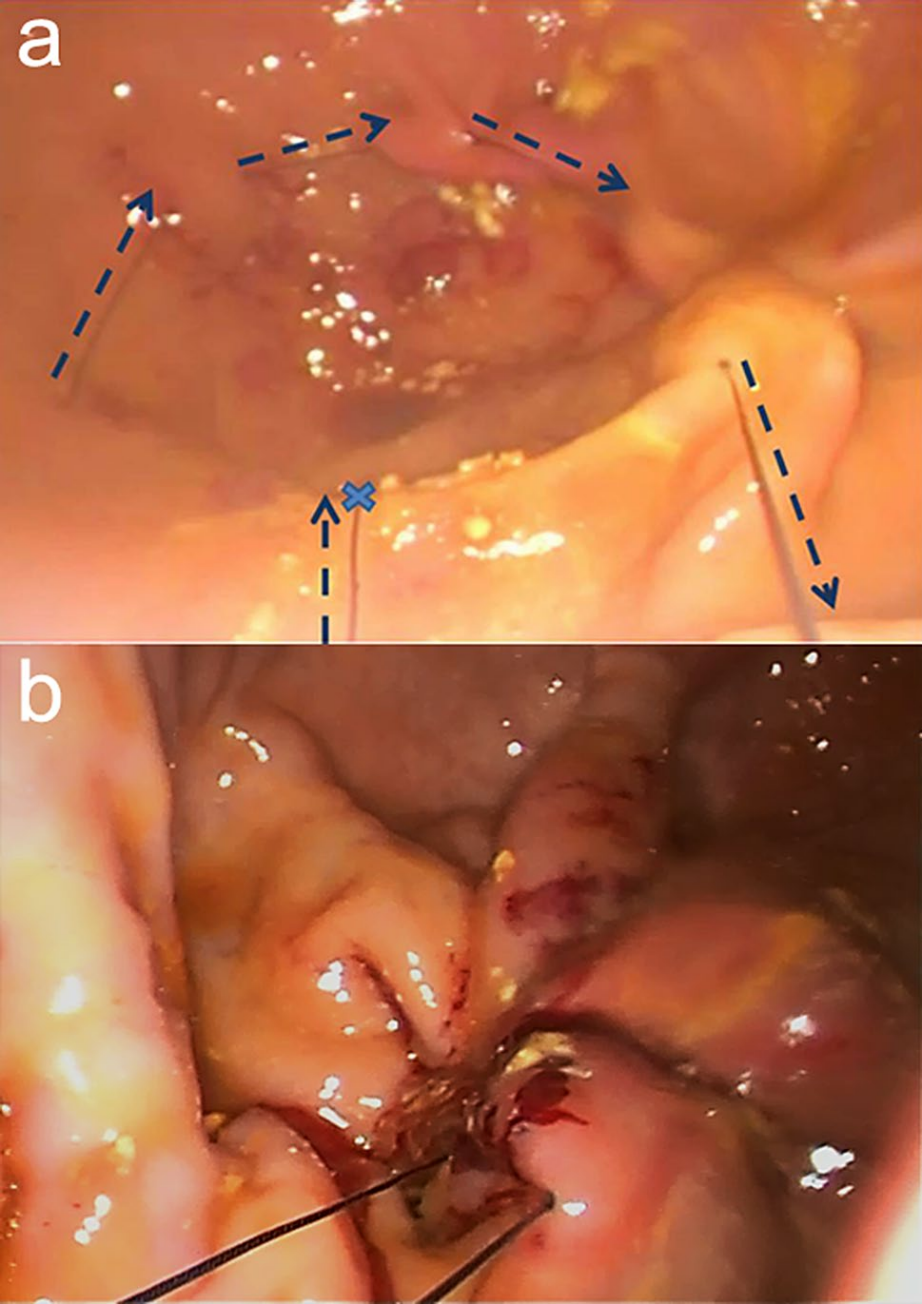
Intragastric plication (**a**) Suture in tobacco pouch (started in the blue xis) and passage of surgical thread (dotted blue arrow) in mucosal and submucosal. (**b**) Final appearance of the suture.

In the G1, the IGS-IGP technique (single-port intragastric and internal suture of stomach, making a longitudinal plication) was performed. Initially, a left pre-umbilical paramedian celiotomy of 2 cm was performed between the cranial and caudal thoracic mammary glands. After accessing the abdominal cavity, the stomach was identified and seized in the body region. Then gastropexy was performed through four equidistant individual sutures with nylon thread 0, followed by gastrostomy. Later, a gel portal system (GelPoint, Applied Med. RS Margarita, CA, USA) with three working portals (5–10 mm) was introduced (Fig. 1). The stomach was inflated with CO_2_ (12 mm Hg), followed by the introduction of a 10 mm rigid 30º endoscope to identify the fundic region of the stomach. Sutures were placed with 2-0 polypropylene thread in a double tobacco pouch pattern. The purpose of this suture was to separate the major curvature to form a gastric sleeve (Fig. 2). At the end, the surgical instruments and the portal were removed, and the stomach closure was performed with 2-0 nylon thread in two suture planes. Skin closure was performed in a conventional manner, in three separate layers^13^.

In G2, exploratory celiotomy was performed, followed by single-port gastroscopy (single-port intragastric). Access to the abdominal cavity and stomach lumen was performed similar to G1. A trocar was positioned and the stomach was inflated. At the end, stomach and skin closure were performed as in G1.

### Postoperative period

A healing spray (Bactrovet Prata®) was used during 12 days. The animals were medicated with 0.4 mg kg^−1^ of meloxicam (Maxicam® 1%) during five days and 15 mg kg^−1^ of sulfadiazine/trimethoprim (Borgal®) during 10 days. The amount and consistency of feed were gradually increased so that the pigs were fed with liquid feed for the first seven days followed by slurry feed for the next seven days. The animals were sacrificed with an anesthetic overdose 18 weeks after surgery.

### Evaluation of fat thickness

**Figure 3:**
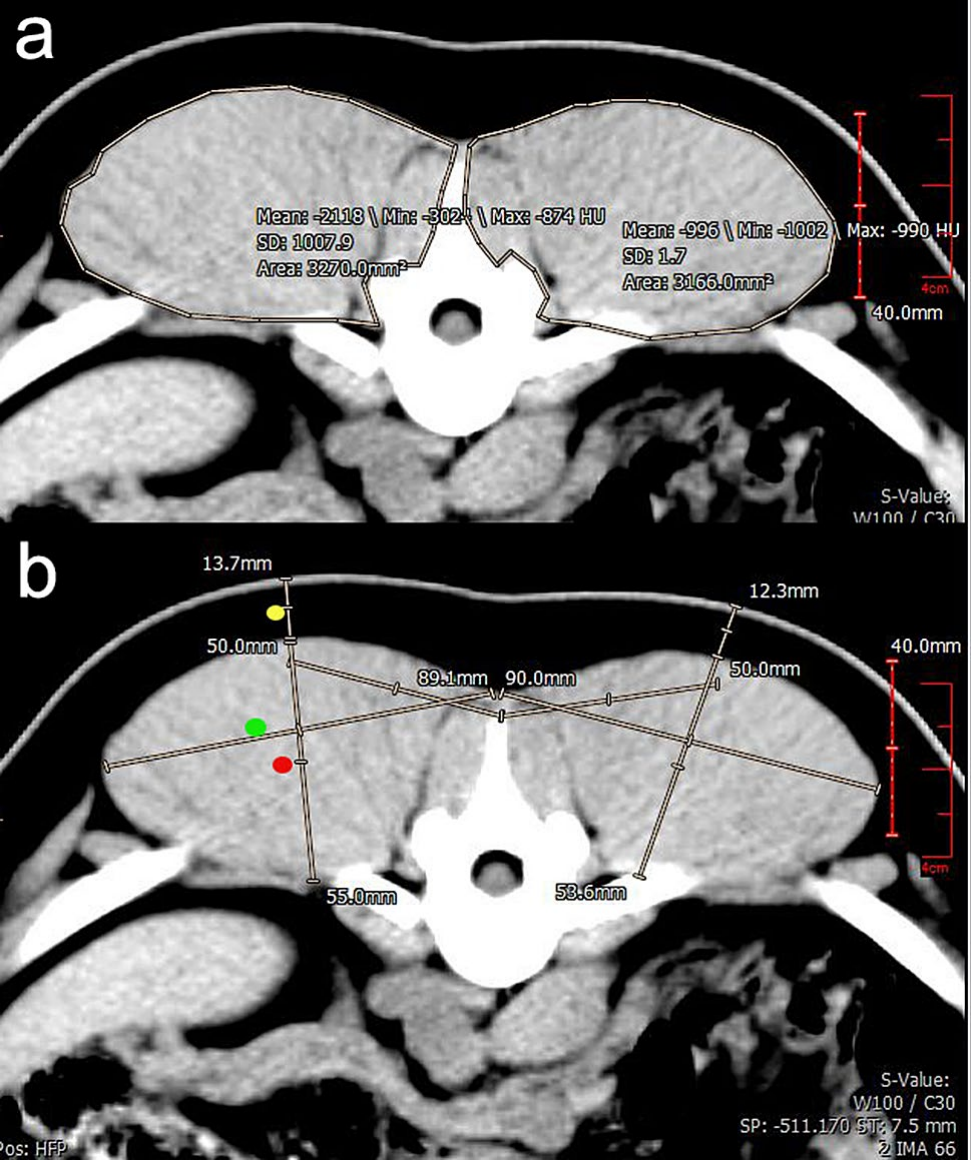
Tomographic images of pigs submitted to single-port intragastric sleeve with transverse section above the last rib. (**a**) Measurement of the loin eye area. (**b**) Measurements of thickness of subcutaneous adipose tissue (yellow dot), depth (red dot) and width (green dot) of longissimus dorsis muscle.

The tomographic images were analyzed using veterinary image processing software (iQ-VIEW 3.0.0), which allowed measurements (above the last rib) of subcutaneous adipose tissue (SAT) and the longissimus dorsi muscle: depth (DLM), width (WLM) and loin eye area (LEA) (Fig. 3).

### Statistical analysis

For statistical analysis, weight gain after the surgical procedure was calculated (current weight—initial weight) and the average weight gain per animal was obtained. The animals were classified into two groups: submitted to single-port intragastric sleeve (G1) and pigs of control group (G2). Data were analyzed using PROC GLM PROC and were submitted to analysis of variance. The means were compared by the SNK test at 5% probability. The Pearson correlation coefficient was used to evaluate the correlation between the variables weight, moment and measures. All analyses were performed using statistical analysis software SAS version 9.2 (SAS Institute, Cary, NC).

### Ethical approval

All procedures performed in studies involving animals were in accordance with the ethical standards of the institution or practice at which the studies were conducted. This article does not contain any studies with human participants performed by any of the authors.

## Results

All animals survived after the procedure, and no postoperative surgical complications were observed. On the seventh postoperative day, all animals had a surgical scar with no signs of infection. The animals recovered uneventfully and remained healthy during the 18 weeks.

**Figure 4:**
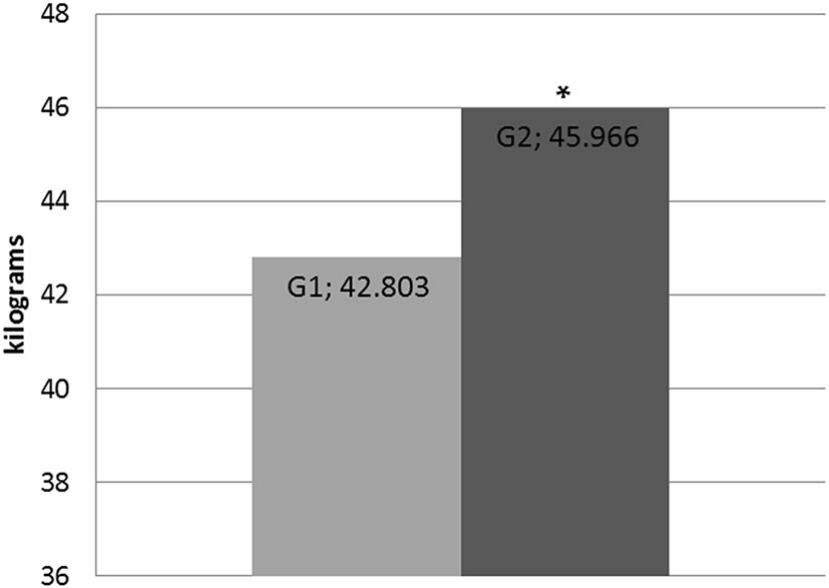
Average of weight gain (kg) in the postoperative period of pigs submitted to single-port intragastric sleeve (G1) and pigs of control group (G2) (*P* ≤ 0.05).

There was a significant difference (*P* < 0.05) in weight gain between groups for the overall period (18 weeks). SHAM pigs (G2) had the greatest weight gain (45.966 ± 4.767 kg), while the weight mean in G1 was 42.803 ± 3.206 kg (Fig. 4).

**Figure 5:**
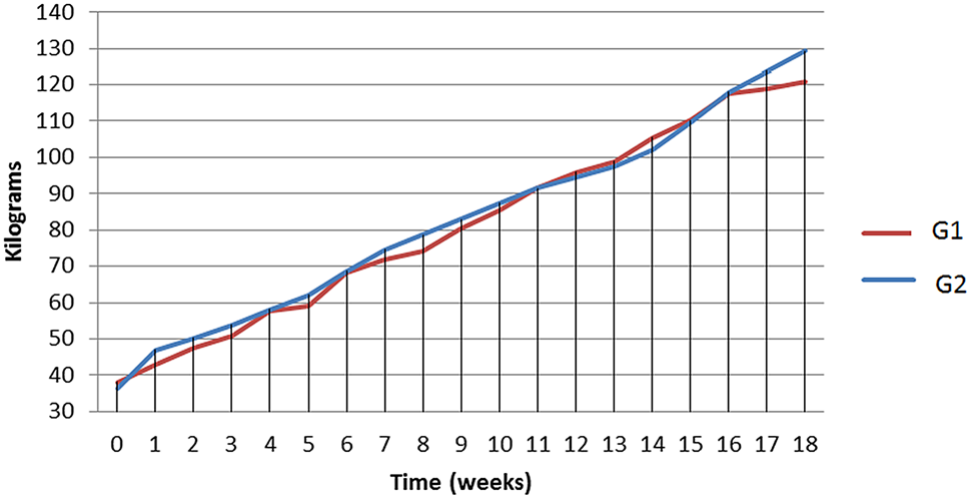
Postoperative weight progression of pigs submitted to single-port intragastric sleeve (G1) and pigs of control group (G2).

Figure 5 shows the curves of the average weight per week until the 18th week of study. G1 remained with the weight mean below G2 until week 10 (85.320 kg and 87.400 kg, respectively). The mean weight were 121.00 kg (G1) and 129.360 kg (G2) at week 18.

The averages of tomographic measurements differed significantly between groups (*P* < 0.05), and G1 obtained the lowest values (Table 1).

**Table 1:** Means and standard deviations of tomographic measurements of carcass of pigs submitted to single-port intragastric sleeve (G1) and pigs of control group (G2).

Measurements	G1		G2	
	Means	SD( ±)	Means	SD ( ±)
WLM (cm)	8.69^b^	0.58	11.10^a^	0.31
DLM (cm)	5.13^b^	0.79	6.29^a^	0.20
SAT (cm)	1.33^b^	0.17	2.36^a^	0.18
LEA (cm^2^)	3.36^b^	0.59	5.24^a^	0.12

DLM, Depth of Longissimus Dorsi muscle; WLM, Width of Longissimus Dorsi muscle;, SAT, Subcutaneous Adipose Tissue; LEA, Loin Eye Area.

^a,b^Means followed by different letters on the same line are statistically different between them (*P* < 0.05) by the SNK test.

The estimated Pearson correlation coefficients are presented in Table 2. All variables analyzed showed significant correlation and high magnitude.

**Table 2:** Pearson correlation coefficients between tomography measurements of in vivo carcass of pigs submitted to single-port intragastric sleeve, weight (P) and moment (M).

	M	W	DLM	WLM	SAT	LEA
M	–					
W	0.96**	–				
DLM	0.90***	0.92**	–			
WLM	0.82**	0.85**	0.95**	–		
SAT	0.82**	0.82**	0.87**	0.94**	–	
LEA	0.85**	0.89**	0.97**	0.99**	0.93**	–

ID, age; M, moment; W, weight; DLM, Depth of Longissimus Dorsi muscle; WLM, Width of Longissimus Dorsi muscle; SAT, Subcutaneous Adipose Tissue; LEA, Loin Eye Area.

*P < 0.05; **P < 0.01.

## Discussion

The new procedure described combines the concepts of intragastric and single portal access, called “hybrid access surgery”^9^. The surgical procedure evaluated was designed to reduce part of the gastric volume, limiting the food intake after the animal undergoes gastric sleeve surgery. The surgeon was able to perform the operative procedure without difficulties in triangulation of the instruments. This difficulty has been reported by some authors^4,14–17^.

The animals recovered well from the surgical procedure, and no complications were observed in transoperative, immediate postoperative and during the follow-up period. Laparoscopic bariatric procedures, although very effective, have complication rates of 3% to 20% and mortality rates of 1%^18,19^. Late post-bariatric complications have been reported after Roux-en-Y with gastric bypass and gastroplasty, including staple line disruption, pocket dilatation, stomach stenosis, anastomotic stenosis and gastro-gastric fistula^20^. One of the main advantages of the single-port intragastric sleeve is its reversibility: in the case of postoperative complications that cannot be tolerated after gastric reduction (dysphagia, vomiting, malnutrition, etc.), endoscopy can be performed to cut intragastric sutures and restore the normal gastric anatomy.

Monitoring of individuals with morbid obesity at baseline and after bariatric surgery requires assessment of body weight and body fat distribution. The means of weight gain differed significantly between groups (*P* < 0.05), intragastric sleeve induced less weight gain than SHAM animals during 18 weekes. Another study evaluated weight gain in growing pigs (70 ± 5 days) in following ileal transposition (IT) when compared with SHAM operated animals after 5 months^21^. Authors observed that SHAM group regained weight faster than IT animals. Similar results were observed in the present study, where G1 maintained weight lower than G2 until week 10. Although IT and IGS-IGP are different bariatric techniques, malabsorptive and restrictive respectively, we believe that IGS-IGP is a less invasive surgical technique, and showed good results with reduced weight gain.

A experimental study compared the effectiveness of a endoluminal gastroplasty in fundus (EGF), sleeve gastroplasty in body (SGB) and SHAM for weight loss in Yorkshire pigs, and observe a significant difference for weight gain among pigs (SGB > EGF > Control, 45.1 > 33.5 > 26.5, *P* < 0.05)^22^. In the present study, the weight gain of G1 pigs (42.8) was lower than SGB pigs.

No weight loss was observed in the animals, since they were followed during the growth and fatterning period (decelerating phase of swine growth curve). The growth period is considered after pigs reach an average of 55–65 kg in a breeding system. However, this period may vary in an experimental model. During the growth period, muscle and lipid tissue gain maintain a 1:1 ratio. In Fig. 5, the curves shows that G1 remained with the weight mean below G2 until week 10 (85.320 kg and 87.400 kg, respectively), and at this week we believe that pigs finished their growth period.

In fatterning period, they consume more food than they need, and the deposition of fat is greater than that of muscle protein. Figure 5 shows that groups presented similar weights between week 11 and 16, so we believe that pigs started the fatterning period after week 11. Then, at the end of the experiment, animals of G2 (129.36 kg) were heavier than animals of G1 (121 kg).

As the objective of the experiment was to evaluate the loss of adipose tissue, the choice of the experimental group in this growth period did not influence the result of the study. The choice of young animals, implies on the ease of handling small animals during experiment and to make a homogeneous groups study belonging to the same litter and age group. Other literatures use young animals for this type of study^21,23,24^.

Currently, research data support the use of bariatric surgery in childhood and adolescence^25,26^. The most common genetic cause of life-threatening obesity in children is the Prader-Willi syndrome, thus bariatric surgery has been an option for these patients^27^. Major concerns exist regarding the safety profile of bariatric procedures in these patients and current techniques present medium and long term complications such as protein-caloric and mineral vitamin malnutrition^25,28^. The decelerating phase of animals may resemble these patients, and IGS-IGP is advantageous because it can be reversed by endoscopy.

Protein deposition in pig carcass is due to energy consumption, and occurs until the genetic limit of muscle deposition is reached. Thereafter, fat mass exceeds the protein deposition^29^. In this work, the excessive carbohydrate consumption was experimentally reproduced by feeding animals ad libitum (G1 and G2) in order to promote accentuated fat deposition in carcass. IT technique performed in growing pigs didn’t reduce protein and fat deposition^21^. While in the present work intragastric sleeve surgery induced a significant (*P* < 0.05) SAT decrease and proteins deposition that was evaluated in CT measurements made in longissumus dorsi muscle.

Although the routine use of CT scans poses health risks and is expensive, this is the reference method for assessing adipose tissue^30^, and for this reason it was chosen in the present study to evaluate the efficacy of the technique. No other study used this method to evaluate the adipose tissue of pigs in post bariatric procedures.

The reduction of superficial fat deposition would be more pronounced if there were used adult animals and with post-surgical food restriction.

## Conclusion

The new intragastric sleeve technique by endoplication appears to be viable, safe and effective, mainly for the reduction of superficial fat deposition in the short term. Additionally, this new technique may become an alternative in bariatric surgical treatment. However, clinical trials regarding the durability of the procedure in humans and long-term adverse effects, mainly related to suturing, are essential to generate advances of this technology.

## Data Availability

All data supporting the findings of this study are available within the paper, Methods and Supplementary Information.
